# Internet-based guided self-help for glioma patients with depressive symptoms: a randomized controlled trial

**DOI:** 10.1007/s11060-017-2712-5

**Published:** 2017-12-13

**Authors:** Florien W. Boele, Martin Klein, Irma M. Verdonck-de Leeuw, Pim Cuijpers, Jan J. Heimans, Tom J. Snijders, Maaike Vos, Ingeborg Bosma, Cees C. Tijssen, Jaap C. Reijneveld, On behalf of the Dutch Society for Neuro-Oncology (LWNO)

**Affiliations:** 1 Department of Medical Psychology, VU University Medical Center, PO Box 7057, 1007 MB Amsterdam, The Netherlands; 2 Department of Neurology, VU University Medical Center, PO Box 7057, 1007 MB Amsterdam, The Netherlands; 3 Department of Otolaryngology – Head & Neck Surgery, VU University Medical Center, PO Box 7057, 1007 MB Amsterdam, The Netherlands; 4 Cancer Center Amsterdam, VU University Medical Center, PO Box 7057, 1007 MB Amsterdam, The Netherlands; 5 Brain Tumor Center Amsterdam, VU University Medical Center, PO Box 7057, 1007 MB Amsterdam, The Netherlands; 6 EMGO Institute for Health and Care Research, VU University Medical Center, PO Box 7057, 1007 MB Amsterdam, The Netherlands; 7 Leeds Institute of Cancer and Pathology, St James’s University Hospital, Leeds, LS9 7TF UK; 8 Clinical Psychology, VU University, Van der Boechorststraat 1, 1081BT Amsterdam, The Netherlands; 9 Brain Center Rudolf Magnus, Department of Neurology, University Medical Center Utrecht, PO Box 85500, 3508 GA Utrecht, The Netherlands; 10 Department of Neurology, Medical Center Haaglanden, PO Box 432, 2501 CK The Hague, The Netherlands; 11 Department of Neurology, University Medical Center Groningen, PO Box 30.0001, 9713 GZ Groningen, The Netherlands; 12 Department of Neurology, St. Elisabeth Hospital, PO Box 90151, 5000 LC Tilburg, The Netherlands

**Keywords:** Glioma, Neuro-oncology, Online therapy, Depression, Fatigue

## Abstract

Depressive symptoms are common in glioma patients, and can negatively affect health-related quality of life (HRQOL). We performed a nation-wide randomized controlled trial to evaluate the effects of an online guided self-help intervention for depressive symptoms in adult glioma patients. Glioma patients with depressive symptoms were randomized to a 5-week online course based on problem-solving therapy, or a waiting list control group. After having received the intervention, the glioma patient groups combined were compared with patients with cancer outside the central nervous system (non-CNS cancer controls), who also received the intervention. Sample size calculations yielded 63 participants to be recruited per arm. The primary outcome [depressive symptoms (CES-D)] and secondary outcomes [fatigue (Checklist Individual Strength (CIS)) and HRQOL (Short Form-36)], were assessed online at baseline, post-intervention, and 3 and 12 months follow-up. In total, 89 glioma patients (intervention *N* = 45; waiting list *N* = 44) and 26 non-CNS cancer controls were included, of whom 35 and 54% completed the intervention, respectively. Recruitment could not be extended beyond 3.5 years due to funding. On depression, no statistically significant differences between the groups were found. Fatigue decreased post-treatment in the glioma intervention group compared with the waiting list group (*p* = 0.054, *d* = 0.306). At 12 months, the physical component summary (HRQOL) remained stable in glioma patients, while scores improved in non-CNS cancer controls (*p* = 0.035, *d* = 0.883). In this underpowered study, no evidence for the effectiveness of online guided self-help for depression or HRQOL in glioma patients was found, but it may improve fatigue.

**Trial registration** Netherlands Trial Register NTR3223.

## Introduction

Following the diagnosis of glioma, many patients experience depressive symptoms. Indeed, systematic reviews and longitudinal studies suggest that ~ 15–20% of glioma patients will develop a depressive disorder during the first 8 months after diagnosis [1, 2]. The increased risk may be maintained up to a year after initial surgery [3]. Depression can have serious negative consequences for glioma patients’ health related quality of life (HRQOL) [4].

A number of tumor- and treatment-related mechanisms, including tumor location [5], elevated intracranial pressure [6], biochemical changes [7], changes in cytokine levels [8], use of antiepileptics [9], and corticosteroids [10] have been suggested to contribute to depression in glioma patients, although the underlying mechanisms are not well understood [7, 11]. Patients’ emotional reactions to the diagnosis and poor prognosis of the disease may contribute considerably [12–14]. Health care professionals may find it difficult to discuss depressive symptoms *especially* when these are understandable [15], leaving depressive symptoms that are potentially treatable, untreated [16].

(Inter)national guidelines suggest that depression in patients with chronic physical conditions should be treated with a combination of medication and psychological treatment such as cognitive behavioral therapy (CBT) [17, 18]. However, a lack of randomized controlled trials (RCTs) in glioma patients makes it difficult to gauge whether these treatment strategies should also be pursued in patients with a brain tumor [19]. Glioma patients are at high risk for cognitive deficits and fatigue, and may struggle to fully benefit from CBT. Antidepressant treatment brings the possibility of adverse drug interactions, introducing a reluctance in both physicians and patients to initiate new pharmaceutical treatment [20].

The present RCT therefore aimed at decreasing depressive symptoms by means of a low-intensity form of CBT [i.e., guided self-help based on problem-solving therapy (PST)], delivered online to increase accessibility and to lower the experienced barrier to mental health care. Internet-based psychological interventions, including PST, have already been found to be equally effective as face-to-face treatment to decrease depressive symptoms in people from the general population [21, 22]. As depression may interact with functional activities and health-related quality of life (HRQOL), the effects of the internet-based therapy on fatigue and overall HRQOL were also evaluated. If proven effective, this online guided self-help intervention could improve psychological care for glioma patients.

## Methods

### Design

This RCT was aimed at evaluating the effects of an internet-based guided self-help intervention targeting depressive symptoms in glioma patients. We compared a group of glioma patients who received the intervention immediately (glioma intervention group; GI group) with a 12 week waiting list control group (glioma waiting list group; GWL group), and with a non-CNS cancer control group (who also receive the intervention; non-CNS cancer control group). A detailed study protocol has been published previously [23], no changes have since been made. The institutional review board of the VU University Medical Center approved the study protocol (registration number 2011/227). The trial was registered in the Netherlands Trial Registry (NTR3223).

### Participants

Between November 2011 and June 2015, patients from 31 hospitals throughout the Netherlands were invited to participate (see Acknowledgments). In each hospital, tailored operating procedures were installed for approaching local patients. Furthermore, advertisements were placed on selected websites, and patient associations helped spread study information. The recruitment period could not be extended due to funding. Data collection was finished in June 2016.

Patients who expressed interest in the study completed online screening questionnaires: the Beck Scale for Suicide Ideation (BSS) [24] and the Center for Epidemiological Studies Depression Scale (CES-D) [25]. Adult (> 18 years of age) glioma patients with WHO grade II, III or IV glioma, and at least mild depressive symptoms (CES-D score ≥ 12) were invited to participate. Similarly, adult (> 18 years of age) patients with non-Hodgkin lymphoma (NHL), chronic lymphatic leukemia (CLL), multiple myeloma (MM), or a myelodysplastic syndrome (MDS) and at least mild depressive symptoms (CES-D score ≥ 12) were invited to participate. Exclusion criteria were (1) no access to the internet and/or no email address; (2) insufficient proficiency of the Dutch language; (3) suicidal intent as screened for with the BSS and followed-up by telephone if needed to check the severity of symptoms. If patients were excluded based on suicidal intent, their primary care physician was informed. All participants provided written informed consent.

### Sample size calculation and randomization

A priori sample size calculations yielded 63 patients to be included per arm (Cohen’s d = 0.50; 1 − β = 0.80, α = 0.05, 25% dropout). A concealed, simple adaptive randomization technique (a folded ticket drawn from a concealed box), was used to allocate glioma patients to the GI or GWL group after baseline assessment. GWL patients could take part in the intervention after a 12-week interval. Patients in the GI group and the non-CNS cancer control group could take part in the intervention directly following baseline assessments. Due to the nature of the study, participants nor researchers could be blind to group allocation.

### Intervention

**Fig. 1:**
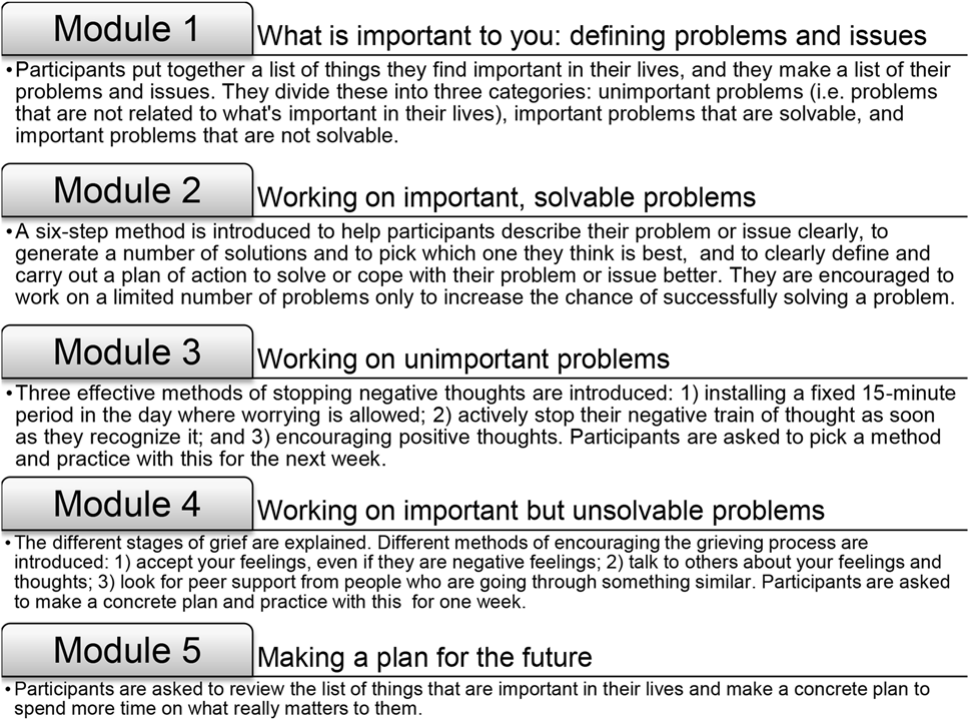
The five modules of the intervention

**Fig. 2:**
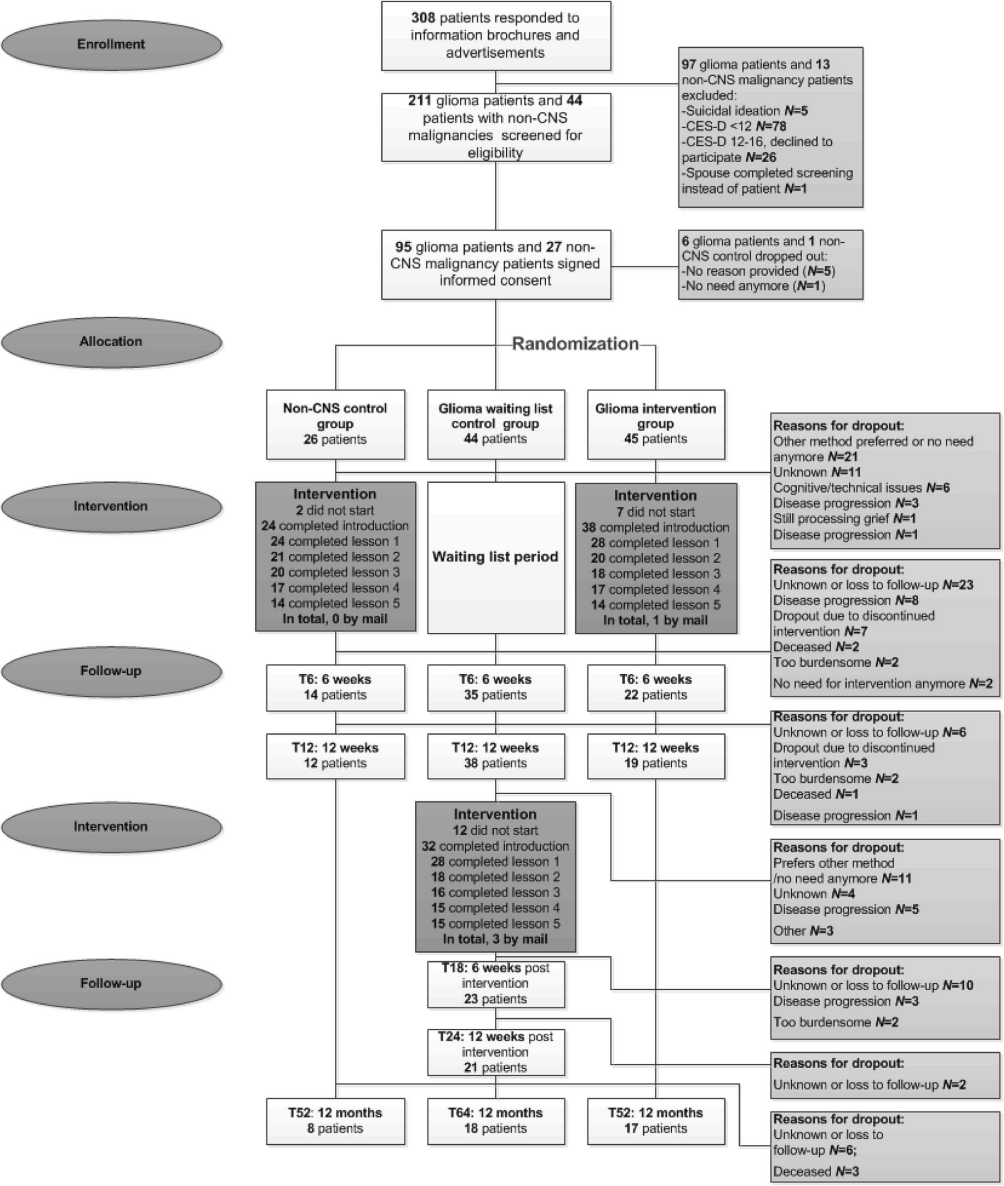
Flow diagram of the study

The intervention was an adaptation of a 5 week online guided self-help course founded on the principles of PST (‘Everything under control’) [26]; disease-specific information and examples were added to the program. The intervention consisted of five modules with examples and exercises (see Fig. 1 and the published protocol [23]). During the intervention, patients described what they felt to be important in their lives, they made a list of their problems and concerns, and worked on improving coping strategies to deal with these issues. Online support from a coach (a researcher-psychologist (FWB), nurse, or a trained and supervised psychology student) was provided to facilitate successful completion of the intervention. This consisted of feedback on completed exercises within 3 working days and additional support on request. Follow-up took place at regular intervals and continued until 12 months after baseline, see Fig. 2. Assessments included patient-reported outcome measures completed online, but could be sent by mail if requested by participants.

### Patient-reported outcomes

#### Primary outcome

##### Depressive symptoms

The change in depressive symptoms as measured with the CES-D [25] at 3 months (GI vs GWL groups) and at 3 and 12 months (total glioma vs non-CNS cancer controls) was the primary outcome measure. This 20-item scale is designed to measure the major components of depressive symptomatology and has good psychometric properties in cancer patient populations [27]. Higher scores indicate more depressive symptoms (range 0–60), with the usual cut-off score for depression set at ≥ 16.

#### Secondary outcomes

##### Fatigue

Fatigue was assessed with the 20-item Checklist Individual Strength (CIS) [28]. Total fatigue scores range from 20 to 140, with higher scores indicating worse functioning. Scores ≥ 76 are indicative of severe fatigue.

##### Health-related quality of life

The Short-Form Health Survey (SF-36) [29] was used to assess HRQOL. The 36 items can be used to calculate two higher-order summary scores which represent physical health (Physical Component Summary; PCS) and mental health (Mental Component Summary; MCS). In a normative sample from the general population, PCS and MCS scores have a mean of 50 with a standard deviation of 10. To assess disease-specific HRQOL in glioma patients, the EORTC Brain Cancer Module (EORTC BN20) [30] was used. Four multi-item scales can be calculated (future uncertainty; visual disorders; motor dysfunctions; communication deficits) and seven single items representing common symptoms. Scale scores range from 0 to 100 with higher scores indicating more symptoms. By error, only 18 of the 20 items of this scale were administered, therefore the motor dysfunction scale and the single item bladder control scores could not be calculated.

##### Cognitive functioning

The six item MOS cognitive functioning scale [31] was used to assess everyday problems in cognitive functioning (range 6–36). Higher scores indicate more cognitive complaints.

##### Use of supportive care

The Trimbos/iMTA questionnaire for Costs associated with Psychiatric Illness (TIC-P) [32] was administered initially with cost effectiveness analysis in mind. Only the questions relating to supportive care use were used.

##### Patient satisfaction

Satisfaction with the intervention was assessed by means of a study specific questionnaire. The perceived usability, readability, the quality of the content and usefulness of the online program, and the coaches’ feedback was assessed.

### Statistical analysis

All analyses were performed using SPSS software version 22. Patient-reported outcomes were transformed into scale scores. ANOVAs and Chi Square tests were performed to compare demographics and clinical variables (age, sex, diagnosis, tumor grade, treatments, disease status, supportive care use, medication, epilepsy, disease-specific symptoms, and cognitive complaints) between the GI and GWL groups. Similarly, age, gender, disease status, and current medication (except use of antiepileptics), were compared between the total glioma group and the non-CNS cancer controls. Chi Square tests and descriptive statistics were used to analyze adherence, reasons for dropout, and patient satisfaction. For descriptive purposes, the percentage of participants scoring above the cut-off for depression (CES-D ≥ 16), fatigue (CIS ≥ 76), and diminished HRQOL (MCS and PCS ≤ 40) was calculated at each time point.

To compare the effects of the intervention to a waiting list only, results from the GI group vs the GWL group at 6 weeks (post intervention) and 12 weeks compared to baseline were analyzed with linear mixed models (LMMs). To compare the effects of the intervention between glioma patients and non-CNS cancer controls, we added the following assessments to form one glioma patient group (‘total glioma group’) that participated in the intervention: T0 (GI group) plus T12 (GWL group); post intervention assessment: T6 (GI group) plus T18 (GWL group); 12 weeks assessment: T12 (GI group) plus T24 (GWL group); 12 months assessment: T52 (GI group) plus T64 (GWL group); see Fig. 2. Results from this total glioma group vs the non-CNS cancer group at post intervention, 12 weeks and 12 months follow-up compared with baseline were also analyzed with LMMs. This statistical method copes well with missing observations due to dropout, therefore missing data were not otherwise imputed. Subjects were added as random effect and interaction, time and group were fixed effects, and a time × group interaction term was used. Toeplitz covariance structures were applied. To correct for baseline differences between the groups, the baseline scores of relevant outcome measures (CES-D score, CIS total fatigue score, SF-36 MCS/PCS) and variables that were significantly different (GI vs GWL group: BN20 future uncertainty scale; total glioma group vs non-CNS cancer group: patient age) were added as covariates. Both intention-to-treat (ITT) and per protocol (PP) analyses were performed for depression, fatigue, and HRQOL (MCS and PCS scores). As it is yet unclear how many modules are required for an effect to be found, all participants who had completed ≥ one module were included in the PP analyses. *P* < 0.05 was considered statistically significant. A Cohen’s d effect size based on the difference in sample means and the pooled pretest standard deviation, corrected for bias, was calculated (0.10–0.29 low; 0.30–0.50 moderate; > 0.50 high) [33, 34].

## Results

### Participants

In total, we received 308 responses through email, phone calls, or (in)complete screening questionnaires, see Fig. 2 for consort flow diagram. Of these, 145 patients could be invited for participation in the study (78.6% glioma, 21.4% non-CNS cancer) and 122 patients agreed to participate (78% glioma, 22% non-CNS cancer). Before baseline assessment, 6 glioma patients and 1 non-CNS cancer patient dropped out. In total, 89 glioma patients were randomized to either the GI group (*N* = 45) or the GWL group (*N* = 44), and 26 non-CNS cancer controls participated.

The majority of patients were women and had middle to high levels of education, see Table 1. Glioma patients most often suffered from a grade II tumor. Approximately 76% of glioma patients were using antiepileptic drugs. GWL patients experienced more uncertainty concerning the future (*M* = 52.1, SD = 21.7) than GI patients (*M* = 42.6, SD = 19.6, *p* = 0.033). No other statistically significant differences were observed.

**Table 1:** Clinical and demographic characteristics of study sample

	Glioma intervention group *N* = 45	Glioma waiting list control group *N* = 44	P value	Glioma total group *N* = 82	Non-CNS control group *N* = 26	P value
Age M (SD)	43.58 (11.69)	46.43 (12.28)	0.265	44.88 (11.97)	52.81 (9.28)	0.003*
Sex N (%)						
Male	19 (42.2%)	18 (40.9%)	0.536	37 (45.1%)	9 (34.6%)	0.345
Female	26 (57.8%)	26 (59.1%)		45 (54.9%)	17 (65.4%)	
Educational level N (%)						
Low	4 (8.9%)	6 (13.6%)	0.734	8 (9.8%)	0 (0%)	0.252
Middle	21 (46.7%)	18 (40.9%)		36 (43.9%)	13 (50.0%)	
High	20 (44.4%)	20 (45.5%)		38 (46.3%)	13 (50.0%)	
Medication use at start of study^b^ N (%)						
Antidepressants	5 (11.1%)	5 (11.4%)	0.970	9 (10.9%)	3 (11.5%)	0.937
Antipsychotics	1 (2.2%)	0 (0%)	0.320	1 (1.2%)	0 (0%)	0.572
Psychostimulants	0 (0%)	0 (0%)	N/a	0 (0%)	1 (3.8%)	0.074
Antiepileptics	35 (77.8%)	33 (75.0%)	0.758	63 (76.8%)	0 (0%)	N/a
Corticosteroids	3 (6.7%)	4 (9.1%)	0.671	6 (7.3%)	3 (11.5%)	0.497
Benzodiazepines	8 (17.8%)	6 (13.6%)	0.592	13 (15.9%)	4 (15.4%)	0.954
Mild opioid analgesics	1 (2.2%)	0 (0%)	0.320	1 (1.2%)	1 (3.8%)	0.387
Disease status during study^c^ N (%)						
Stabile disease or remission	29 (64.4%)	31 (70.5%)	0.600	56 (68.3%)	11 (42.3%)	0.278
Disease progression	6 (13.3%)	6 (13.6%)		10 (12.2%)	2 (7.7%)	
Active treatment	10 (22.2%)	6 (13.6%)		15 (18.3%)	7 (26.9%)	
Other support in the past 4 weeks (at baseline) N (%)						
Primary care physician	17 (37.8%)	19 (43.2%)	0.669	36 (43.9%)	11 (42.3%)	1.000
Psychologist, psychiatrist, or counsellor	13 (28.9%)	11 (25%)	0.813	24 (29.3%)	11 (42.3%)	0.155
Social worker	3 (6.7%)	5 (11.4%)	0.479	8 (9.8%)	3 (11.5%)	0.711
Alcohol/drugs coach	1 (2.2%)	0 (0%)	1.000	1 (1.2%)	0 (0%)	1.000
Self-help group	1 (2.2%)	5 (11.4%)	0.106	6 (7.3%)	3 (11.5%)	0.424
Company physician	10 (22.2%)	6 (13.6%)	0.410	16 (19.5%)	4 (15.4%)	1.000
Physiotherapist	10 (22.2%)	9 (20.5%)	1.000	19 (23.2%)	7 (26.9%)	0.599
Alternative healer	5 (11.1%)	5 (11.4%)	1.000	10 (12.2%)	6 (23.1%)	0.194
Tumor type^a^ N (%)						
Pontine glioma	0 (0%)	1 (2.3%)	0.707			
Ganglioglioma	1 (2.2%)	0 (0%)				
Astrocytoma	21 (46.7%)	17 (38.6%)				
Oligodendroglioma	10 (22.2%)	11 (25.0%)				
Oligoastrocytoma	6 (13.3%)	7 (15.9%)				
Glioblastoma	6 (13.3%)	7 (15.9%)				
Unspecified glioma	1 (2.2%)	0 (0%)				
Meningioma	0 (0%)	1 (2.3%)				
Glioma grade N (%)						
Grade II	26 (57.8%)	23 (52.3%)	0.898			
Grade III	13 (28.9%)	13 (29.5%)				
Grade IV	6 (13.3%)	7 (15.9%)				
Epilepsy N (%)						
Yes	23 (51.1%)	23 (52.3%)	0.913			
No	22 (48.9%)	21 (47.7%)				
Type of surgery N (%)						
None	0 (0%)	1 (2.3%)	0.168			
Biopsy	4 (8.9%)	9 (20.5%)				
Resection	41 (91.1%)	34 (77.3%)				
Treatments received at start of study N (%)						
Radiation therapy	30 (66.7%)	29 (65.9%)	0.940			
Chemotherapy	18 (40.0%)	24 (54.5%)	0.169			
Cognitive complaints M (SD)	24.7 (6.6)	22.6 (5.9)	0.122			
Disease-specific symptoms^d^ M (SD)						
Future uncertainty	42.6 (19.6)	52.1 (21.7)	0.033*			
Visual disorder	19.8 (20.4)	21.0 (19.8)	0.778			
Communication deficits	26.2 (23.0)	30.6 (23.9)	0.380			
Headaches	28.9 (27.2)	33.3 (33.7)	0.495			
Seizures	10.4 (21.1)	16.7 (24.4)	0.196			
Drowsiness	26.7 (27.2)	36.4 (28.6)	0.104			
Bothered by hair loss	11.9 (21.5)	17.4 (30.9)	0.325			
Bothered by itching skin	19.3 (27.1)	19.7 (27.2)	0.940			
Weakness of legs	10.4 (21.1)	10.6 (20.0)	0.957			

*p < 0.05

^a^One patient was diagnosed with a meningioma, this was discovered after randomization

^b^Other medication includes treatment for arthritis, antivirals, antibiotics, antimyotics, antihypertensives, cholesterol inhibitors, anticoagulants, non-opioid analgesics, antiemetics, antihistamines, stomach protectors, thyroid medication, antidiabetic agents, drugs for bowel and bladder function, calcium and vitamin supplements

^c^Disease status missing in one patient (glioma waiting list group)

^d^Two items of the EORTC QLQ BN20 were not administered by error; motor dysfunction and bladder control scores are missing

Most non-CNS cancer controls were diagnosed with a non-Hodgkin lymphoma (46.2%), other diagnoses were chronic lymphatic leukemia (11.5%), multiple myeloma (11.5%) and myelodysplastic syndrome (11.5%). Non-CNS cancer controls were older than patients with glioma (*M* = 52.8, SD = 9.3 vs *M* = 45.0, SD = 11.9 *p* = 0.003); no other statistically significant differences between the groups were observed. Outside hospital appointments, patients in all groups reported visiting their primary care physician, company physician, physiotherapist, psychologist/psychiatrist/counsellor, social worker, substance abuse coach, alternative healer, and self-help group (no statistically significant differences, see Table 1).

### Effects of the intervention on depression, fatigue and HRQOL

**Fig. 3:**
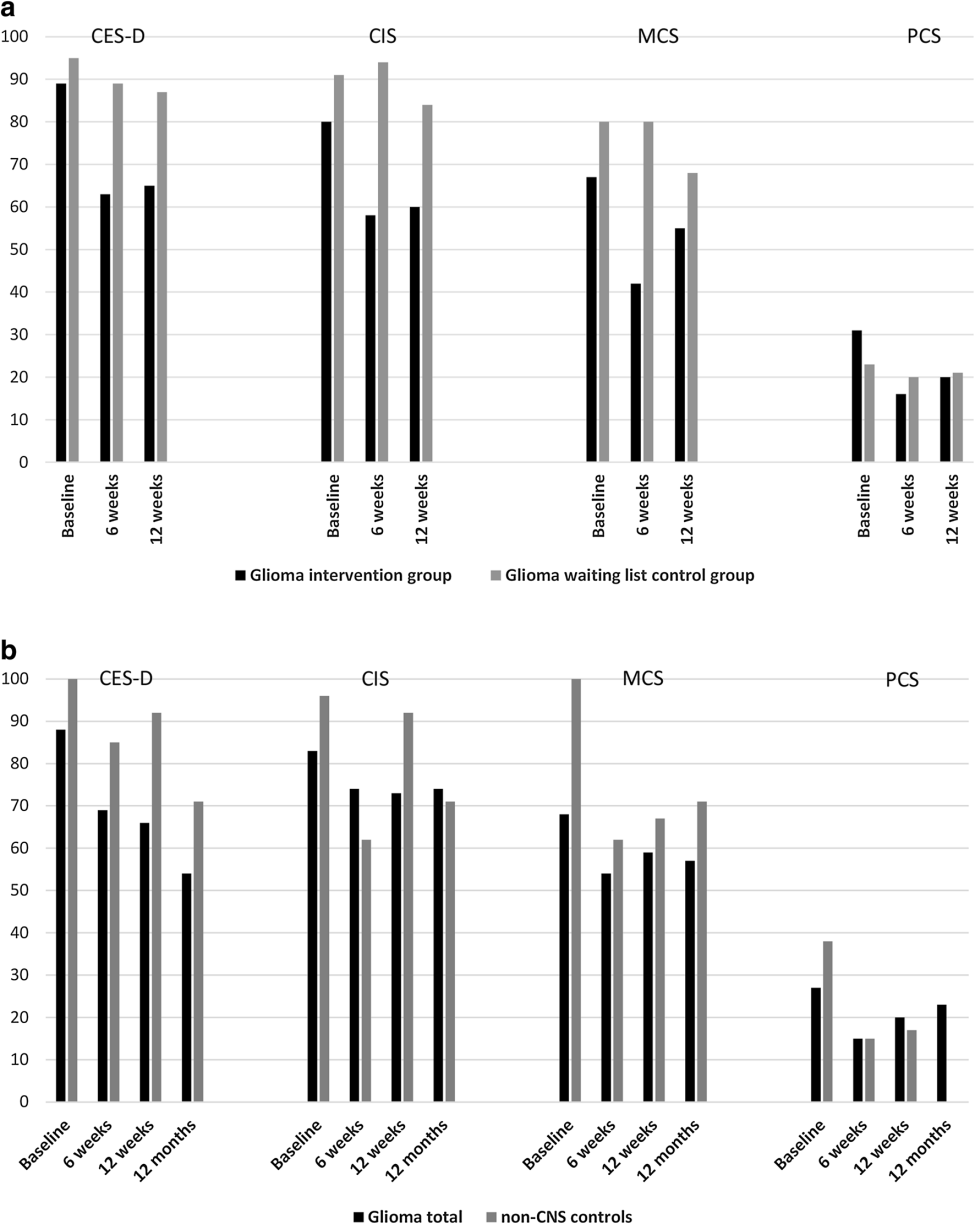
Percentages of patients scoring above the cut-off for depression (CES-D ≥ 16), fatigue (CIS ≥ 76), and diminished HRQOL (MCS and PCS ≤ 40). **a** Glioma intervention group and glioma waiting list control group. **b** Total glioma group and non-CNS cancer control group. *CES-D* Center for Epidemiological Studies—Depression Scale, *CIS* Checklist Individual Strength, *MCS* Short-Form 36 Health Survey Mental Component Summary, *PCS* Short-Form 36 Health Survey Physical Component Summary

Figure 3a, b illustrate the percentage of patients scoring above the cut-off for depression, fatigue, and diminished HRQOL at the different time points.

#### Glioma intervention group vs. glioma waiting list control group

No statistically significant differences in depression scores were found between the GI group and GWL group (Table 2).

**Table 2:** Results of analyses comparing the glioma intervention group with the glioma waiting list control group

	Intention to treat			Per protocol		
	Glioma intervention group	Glioma waiting list control group	P value, ES, 95% CI	Glioma intervention group	Glioma waiting list control group	P value, ES, 95% CI
Depression (CES-D) M (SD)						
Baseline	*N* = 45 21.96 (5.9)	*N* = 43 24.98 (6.9)		*N* = 35 21.51 (6.1)	*N* = 28 24.07 (6.6)	
After intervention	*N* = 19 18.84 (6.4)	*N* = 34 23.09 (7.1)	*p* = 0.390, ES = 0.190, 95% CI = − 4.49 to 2.67	*N* = 18 19.61 (5.7)	*N* = 26 23.50 (6.1)	*p* = 0.454, ES = 0.038, 95% CI = − 6.17 to 2.79
After 12 weeks	*N* = 19 19.63 (7.5)	*N* = 37 22.38 (6.3)	*p* = 0.614, ES = 0.042, 95% CI = − 2.67 to 4.49	*N* = 18 19.89 (7.6)	*N* = 28 22.86 (5.7)	*p* = 0.954, ES = 0.012, 95% CI = − 3.82 to 4.05
Fatigue (CIS) M (SD)						
Baseline	*N* = 45 88.27 (22.0)	*N* = 43 99.81 (18.4)		*N* = 35 91.06 (20.5)	*N* = 28 101.71 (18.4)	
After intervention	*N* = 19 81.58 (25.8)	*N* = 33 99.39 (16.3)	*p* = 0.054* ES = 0.306, 95% CI = − 17.63 to 0.15	*N* = 18 83.94 (24.3)	*N* = 25 101.08 (16.2)	*p* = 0.112, ES = 0.327, 95% CI = − 18.13 to 1.93
After 12 weeks	*N* = 19 80.16 (27.0)	*N* = 37 96.00 (17.3)	*p* = 0.238, ES = 0.210, 95% CI = − 3.85 to 15.25	*N* = 18 79.94 (27.8)	*N* = 28 96.50 (16.5)	*p* = 0.310, ES = 0.298, 95% CI = − 5.35 to 16.59
Health-related quality of life (SF-36 MCS), M (SD)						
Baseline	*N* = 45 36.71 (7.9)	*N* = 44 32.74 (9.4)		*N* = 35 36.99 (7.7)	*N* = 28 31.79 (10.4)	
After intervention	*N* = 19 38.34 (10.3)	*N* = 34 32.97 (8.9)	*p* = 0.326, ES = 0.159, 95% CI = − 2.08 to 6.20	*N* = 18 37.78 (10.3)	*N* = 26 31.38 (8.5)	*p* = 0.349, ES = 0.132, 95% CI = − 2.50 to 6.99
After 12 weeks	*N* = 19 40.23 (9.6)	*N* = 37 33.53 (9.9)	*p* = 0.433, ES = 0.310, 95% CI = − 6.32 to 2.73	*N* = 18 40.56 (9.8)	*N* = 28 32.4 (8.9)	*p* = 0.431, ES = 0.325, 95% CI = − 7.24 to 3.13
Health-related quality of life (SF-36 PCS), M (SD)						
Baseline	*N* = 45 47.63 (10.9)	*N* = 44 45.55 (9.1)		*N* = 35 46.88 (11.1)	*N* = 28 46.82 (9.5)	
After intervention	*N* = 19 51.17 (11.4)	*N* = 34 46.95 (10.1)	*p* = 0.141, ES = 0.211, 95% CI = − 1.07 to 7.36	*N* = 18 51.07 (11.7)	*N* = 26 48.47 (10.5)	*p* = 0.347, ES = 0.239, 95% CI = − 2.48 to 6.98
After 12 weeks	*N* = 19 48.57 (9.8)	*N* = 37 47.25 (10.7)	*p* = 0.993, ES = 0.075, 95% CI = − 4.01 to 4.04	*N* = 18 48.16 (9.9)	*N* = 28 47.43 (11.5)	*p* = 0.882, ES = 0.063, 95% CI = − 5.08 to 4.38

*CES-D* Center for Epidemiological Studies—Depression Scale, *CIS* Checklist Individual Strength, *ES* effect size, *ITT* Intention to treat, *PP* per protocol, *SF-36 MCS* Short-Form 36 Health Survey Mental Component Summary, *SF-36 PCS* Short-Form 36 Health Survey Physical Component Summary

* *p* < 0.05

Fatigue scores decreased between baseline and 6 weeks (post-intervention) in the GI group compared with the GWL group (*p* = 0.054, *d* = 0.306; intention to treat analysis only). No other statistically significant results were found when comparing the GI group and the GWL control group.

#### Total glioma group vs. non-CNS control group

No statistically significant differences in depression scores were found between the total glioma group and the non-CNS cancer group (Table 3).

**Table 3:** Results of analyses comparing the total glioma group with the non-CNS control group

	Intention to treat			Per protocol		
	Glioma total group	Non-CNS control group	P value, ES, 95% CI	Glioma total group	Non-CNS control group	P value, ES, 95% CI
Depression (CES-D) M (SD)						
Baseline	*N* = 82 22.14 (6.1)	*N* = 26 25.08 (6.6)		*N* = 63 22.11 (5.9)	*N* = 24 25.08 (6.7)	
After intervention	*N* = 42 19.52 (7.5)	*N* = 13 20.31 (6.3)	*p* = 0.267, ES = 0.300 95% CI = − 1.70 to 6.09	*N* = 38 19.82 (6.9)	*N* = 13 20.31 (6.3)	*p* = 0.272, ES = 0.401, 95% CI = − 1.75 to 6.18
After 12 weeks	*N* = 41 20.85 (8.5)	*N* = 12 25.41 (7.1)	*p* = 0.467, ES = 0.302, 95% CI = − 6.36 to 2.93	*N* = 38 20.42 (8.4)	*N* = 12 25.42 (7.1)	*p* = 0.418, ES = 0.218, 95% CI = − 6.66 to 2.78
After 12 months	*N* = 35 18.60 (9.3)	*N* = 7 18.14 (7.0)	*p* = 0.390, ES = 0.499, 95% CI = − 3.31 to 8.42	*N* = 33 17.85 (9.0)	*N* = 7 18.14 (6.9)	*p* = 0.447, ES = 0.433, 95% CI = − 3.76 to 8.00
Fatigue (CIS) M (SD)						
Baseline	*N* = 82 91.76 (20.2)	*N* = 26 96.85 (13.5)		*N* = 63 93.48 (18.9)	*N* = 24 96.75 (13.0)	
After intervention	*N* = 42 89.74 (24.9)	*N* = 13 86.74 (18.1)	*p* = 0.208, ES = 0.426, 95% CI = − 3.59 to 16.35	*N* = 38 91.02 (23.5)	*N* = 13 86.69 (18.1)	*p* = 0.241, ES = 0.430, 95% CI = − 4.06 to 16.00
After 12 weeks	*N* = 41 90.12 (27.2)	*N* = 12 90.42 (15.2)	*p* = 0.503, ES = 0.252, 95% CI = − 8.16 to 16.54	*N* = 38 88.53 (27.1)	*N* = 12 90.42 (15.2)	*p* = 0.716, ES = 0.078, 95% CI = − 10.12 to 14.69
After 12 months	*N* = 34 87.94 (24.2)	*N* = 7 94.57 (23.7)	*p* = 0.587, ES = 0.081, 95% CI = − 20.69 to 11.78	*N* = 32 86.19 (23.9)	*N* = 7 94.57 (23.7)	*p* = 0.438, ES = 0.289, 95% CI = − 22.60 to 9.86
Health-related quality of life (SF-36 MCS) M (SD)						
Baseline	*N* = 82 35.27 (9.0)	*N* = 26 29.73 (7.3)		*N* = 63 34.94 (8.5)	*N* = 24 29.58 (7.2)	
After intervention	*N* = 41 36.79 (10.2)	*N* = 13 35.20 (8.3)	*p* = 0.159, ES = 0.455, 95% CI = − 8.43 to 1.40	*N* = 37 36.53 (9.9)	*N* = 13 35.20 (8.3)	*p* = 0.201, ES = 0.489, 95% CI = − 8.10 to 1.72
After 12 weeks	*N* = 41 37.42 (10.2)	*N* = 12 33.48 (13.0)	*p* = 0.385, ES = 0.184, 95% CI = − 7.50 to 2.92	*N* = 38 37.92 (10.2)	*N* = 12 33.48 (13.0)	*p* = 0.478, ES = 0.112, 95% CI = − 7.26 to 3.42
After 12 months	*N* = 35 39.83 (9.2)	*N* = 7 33.34 (10.4)	*p* = 0.601, ES = 0.109, 95% CI = – 5.12 to 8.80	*N* = 33 40.24 (9.3)	*N* = 7 33.34 (10.4)	*p* = 0.545, ES = 0.187, 95% CI = − 4.89 to 9.19
Health-related quality of life (SF-36 PCS) M (SD)						
Baseline	*N* = 82 47.46 (10.7)	*N* = 26 44.44 (9.6)		*N* = 63 47.13 (11.2)	*N* = 24 44.52 (9.9)	
After intervention	*N* = 41 50.05 (10.7)	*N* = 13 47.81 (11.7)	*p* = 0.734, ES = 0.074, 95% CI = − 5.24 to 3.70	*N* = 37 50.06 (11.24)	*N* = 13 47.81 (11.7)	*p* = 0.822, ES = 0.033, 95% CI = − 4.93 to 3.92
After 12 weeks	*N* = 41 48.41 (9.9)	*N* = 12 49.07 (11.4)	*p* = 0.549, ES = 0.350, 95% CI= – 6.76 to 3.62	*N* = 38 48.66 (9.6)	*N* = 12 49.07 (11.4)	*p* = 0.662, ES = 0.276, 95% CI = − 6.80 to 4.35
After 12 months	*N* = 35 47.61 (11.3)	*N* = 7 53.88 (7.9)	*p* = 0.035*, ES = 0.883, 95% CI = − 13.08 to − 0.49	*N* = 33 48.32 (11.0)	*N* = 7 53.86 (7.9)	*p* = 0.053*, ES = 0.744, 95% CI = – 13.09 to 0.08

*CES-D* Center for Epidemiological Studies—Depression Scale, *CIS* Checklist Individual Strength, *ES* effect size, *ITT* intention to treat, *PP* per protocol, *SF-36 MCS* Short-Form 36 Health Survey Mental Component Summary, *SF-36 PCS* Short-Form 36 Health Survey Physical Component Summary

* *p* < 0.05

Both intention to treat and per protocol analysis yielded statistically significant change with a large effect size in the PCS score between baseline and 12 months follow-up (ITT: *p* = 0.035, *d* = 0.883; PP: *p* = 0.053; *d* = 0. 744) with scores remaining stable in glioma patients, while improving in non-CNS cancer controls. No other statistically significant results were found between the total glioma group and the non-CNS cancer control group.

### Intervention adherence and satisfaction

Adherence to the intervention was lower in the total glioma patient group (*N* = 82) compared to non-CNS cancer controls (*p* = 0.043). In glioma patients, intervention adherence was 85% for the introduction and 77, 52, 40, 37 and 35% for modules 1 through 5, respectively. In non-CNS cancer controls, intervention adherence was 92% for the introduction and 92, 81, 73, 65, and 54% for modules 1 through 5, respectively.

Reasons reported for not completing the program did not differ between the groups (*p* > 0.05). The most common reasons were: course did not meet their needs, no need for the program anymore, or different kind of treatment preferred (44% glioma; 58% non-CNS); no reason provided (28% glioma; 25% non-CNS); disease progression (11% glioma; 8% non-CNS); and cognitive/technical difficulties (13% glioma only). Other reasons were: too burdensome (2% glioma); wrong timing (2% glioma); wanted to continue working on module 4 instead (2% non-CNS).

The patient satisfaction questionnaire was completed by 37/82 glioma patients (of whom 62% had completed the intervention) and 12/26 non-CNS cancer controls (of whom 75% had completed the intervention). Most patients said they had benefitted from participating (73% glioma; 67% non-CNS), and that they thought the program was useful (92% in both groups) and informative (86% glioma; 92% non-CNS). The program’s content (78% glioma; 75% non-CNS) and readability (88% glioma; 92% non-CNS) were rated good to very good. Feedback from the coach was considered to be useful (81% glioma; 75% non-CNS). However, the majority of patients indicated that they did not believe their depressive symptoms had reduced after the online program (57% glioma; 67% non-CNS).

## Discussion

Contrary to our expectations there were no beneficial effects of the intervention on depressive symptoms in our sample of glioma patients with depressive symptoms. Similarly, we found no changes in depressive symptoms in non-CNS cancer controls, which may indicate that the lack of effect is not likely the result of disease-specific issues. We did find a moderate effect on fatigue when comparing the glioma intervention group with the waiting list control group. This indicates that the program may help patients tackle their fatigue—at least in the short term, as the effect was no longer observed after 12 weeks follow-up. Nevertheless, this finding is important as fatigue is one of the most commonly reported and debilitating symptoms in glioma [35, 36], and little evidence for effective interventions exists [37]. Of note, this effect did not hold in PP analyses, possibly due to the smaller sample size leading to poorer statistical power.

Unexpectedly, at 12 months follow-up the physical component of HRQOL remained stable in glioma patients, while scores improved in non-CNS cancer controls. Both this finding and the borderline significant effect on fatigue could, in part, be explained by a bias due to participant dropout. As only 31% of non-CNS cancer controls and ~ 39% of glioma patients completed the 12 month assessments, it seems possible that those with worse physical HRQOL discontinued study participation, leading to an overestimation of HRQOL—a known issue in glioma studies [38] which could apply to non-CNS cancer groups as well.

We encountered great difficulties regarding recruitment, attrition, and adherence. Despite nation-wide recruitment efforts spanning a 3.5 years period, only 308 patients responded to the study information of whom 40% could be recruited. This is a low participation rate considering that in general, 60% of cancer patients participate in intervention studies to reduce distress [39]. The final sample consisted of only ~ 71 and ~ 41% of the required sample size for glioma patients and non-CNS cancer controls, respectively. Moreover, attrition was higher than anticipated with previous studies reporting ~ 31–50% dropout [40, 41]. Adherence to the intervention was low, which is a common problem in (internet-based) psychological intervention studies [40, 42].

We aimed to perform this RCT with high external validity, using few exclusion criteria. Patients were allowed to use antidepressants and/or mental health care services. Although there were no statistically significant differences between study arms, about 25–29% of glioma patients and 42% of non-CNS cancer controls used other mental health care services (e.g., psychologist, psychiatrist, or counsellor). This may have attenuated possible effects of the intervention. Over half of our glioma patient sample had a low-grade tumor. While there is no reason to assume depressive symptoms might differ between those with low- or high-grade tumors, high-grade gliomas are more common and patients with higher grade tumors tend to have a different disease burden [43]. Our study sample may therefore not be a completely accurate representation of the general glioma patient population. Moreover, due to the nature of the intervention we do not know whether patients required any help from their family caregiver to complete the program.

Despite the study’s shortcomings, it is the first RCT to explore the effectiveness of online psychological treatment in glioma patients. Similar studies in other neurological/oncological patient groups yielded mixed results. In a small sample of patients with chronic spinal cord injury, an online CBT-based program reduced distress in both the intervention group and a waiting list control group, but no difference was found between the groups [44]. Other supportive internet-based interventions have shown positive effects on HRQOL in cancer patients [45]. Especially programs based on CBT appear to be effective in alleviating depressive symptoms in patients with chronic illness [46, 47]. As PST is a low-intensity form of CBT, a more intensive treatment might be required to effectively treat depressive symptoms in glioma patients. The internet-based program might still be useful as a part of stepped care, where interventions of increased intensity are introduced step by step. Indeed, this concept has been found effective in other cancer populations [48, 49]. However intensive therapies may not be feasible for all glioma patients, in particular those with more cognitive deficits and fatigue, hence other avenues for support should be explored as well.

To conclude, this RCT showed that in glioma patients, a guided internet-based PST is not effective in reducing depressive symptoms or improving HRQOL, but it seems to have a positive effect on fatigue. In part, the lack of statistically significant effects could be explained by poor statistical power, low adherence, and high attrition rates. Further research is required to determine effective treatments for depressive symptoms in glioma patients.
